# Cooperative Location Method for Leader-Follower UAV Formation Based on Follower UAV’s Moving Vector

**DOI:** 10.3390/s22197125

**Published:** 2022-09-20

**Authors:** Xudong Zhu, Jizhou Lai, Sheng Chen

**Affiliations:** College of Automation Engineering, Nanjing University of Aeronautics and Astronautics, Nanjing 210016, China

**Keywords:** cooperative positioning, minimum cooperative unit, moving vector, Markov chain

## Abstract

The traditional leader-follower Unmanned Aerial Vehicle (UAV) formation cooperative positioning (CP) algorithm, based on relative ranging, requires at least four leader UAV positions to be known accurately, using relative distance with leader UAVs to achieve the unknown position follower UAV’s high-precision positioning. When the number of the known position leader UAVs is limited, the traditional CP algorithm is not applicable. Aiming at the minimum cooperative unit, which consists of a known position leader UAV and an unknown position follower UAV, this paper proposes a CP method based on the follower UAV’s moving vector. Considering the follower UAV can only acquire the single distance with the leader UAV at each distance-sampling period, it is difficult to determine the follower UAV’s spatial location. The follower UAV’s moving vector is used to construct position observation of the follower UAV’s inertial navigation system (INS). High-precision positioning is achieved by combining the follower UAV’s moving vector. In the process of CP, the leader UAV obtains a high-precision position by an INS/Global Positioning System (GPS) loosely integrated navigation system and transmits its position information to the follower UAV. Based on accurate modeling of the follower UAV’s INS, the position, velocity and heading observation equation of the follower UAV’s INS are constructed. The improved extended Kalman filtering is designed to estimate the state vector to improve the follower UAV’s positioning accuracy. In addition, considering that the datalink system based on radio signals may be interfered with by the external environment, it is difficult for the follower UAV to obtain relative distance information from the leader UAV in real time. In this paper, the availability of the relative distance information is judged by a two-state Markov chain. Finally, a real flight test is conducted to validate the performance of the proposed algorithm.

## 1. Introduction

Over the past few decades, UAVs have been widely used in military and civilian fields. In the civilian field, they are mainly used for rescue missions [1], border patrol [2] and formation flying [3]. In the military field, they are mainly used for coordinated strikes and coordinated tracking [4]. However, due to the constraints of the volume and load of UAVs, a single UAV has low success rates in performing tasks. Multiple UAVs formation can make full use of their resource advantage, and the efficiency of performing tasks is much greater than a single UAV. However, multiple UAVs’ autonomous flight needs to solve two basic problems: firstly, solving the problem of UAVs’ real-time navigation and positioning. Secondly, solving the problem of UAVs’ real-time tracking control, mission and trajectory planning. The high-precision navigation information is the input of the UAV flight control system, so how to accurately acquire the UAVs’ high-precision navigation information is the key for a UAV formation to complete a flight mission.

INS is an autonomous, independent dead-reckoning system, that has been widely used in UAVs. However, the navigation accuracy is difficult to guarantee when only using the INS due to inertial measurement unit’s (IMU) error accumulation. When the satellite signals are available, the GPS receiver can provide the long-term reliable position information for the UAV. Thus, the INS/GPS loosely integrated navigation system is widely used in UAV formation [5,6,7]. In addition, the original information output from the satellite receiver, such as pseudo-range and carrier phase, can be used as system observation to correct INS’s long-term cumulative error. W Khalaf proposed an INS/GPS adaptive, tightly integrated method by fusing the GPS original pseudo-range, magnetometer and barometric altimeter information [8]. S Huang proposed an INS/GPS integrated navigation method based on GPS dual antennas. In this method, the two-difference calculation is performed by carrier phase measurement. The two-difference observation equation is established to improve the UAV’s attitude accuracy [9].

However, satellite signals may be interfered with by the external environment. Abundant external relative perception information among UAVs can effectively improve UAVs’ positioning accuracy. The airborne datalink system, as a communication system based on radio data transmission and receiving, is widely used in the field of collaborative navigation [10]. Under the condition of a UAV cooperative network, the high-precision time reference is established through a datalink time precision alignment method [11]. The relative distance, azimuth and pitch information between UAVs can be provided in real time by Time of Arrival (TOA) and Angle of Arrival (AOA) methods [12]. To the best of our knowledge, the datalink system can provide a relative ranging accuracy of about 30m within the 550 km communication range, under the condition of a time-synchronized cooperative network. Aiming at the relative ranging mode of the datalink system, many researchers have carried out a lot of works to realize multiple UAVs’ high-precision CP. Yang proposed a multi-UAVs collaborative navigation method based on relative distance and magnetic sensor measurement [13]. In this method, the UAV’s kinematic model is constructed combining the output of a magnetic sensor. The accumulated error of the dead-reckoning system is corrected using distance observation. Qu Y [14] proposed a CP method of low-cost UAV using relative range measurements in multi-UAVs flight. Under the condition that at least four leader UAV positions are known in the UAV formation, the relative distance with the leader UAVs is used as the system observation to improve the unknown position follower UAV’s positioning accuracy. In addition, Chen M [15] proposed a cooperative navigation method for a UAV swarm based on cooperative dilution of precision. The spatial configuration between leader UAVs and follower UAVs is considered, using relative distance with four known position leader UAVs in the optimal spatial geometry configuration as the observation method to further improve follower UAVs’ positioning accuracy. Through the above analysis, the current mainstream CP algorithm based on relative distance mainly adopts the multiple leader UAV CP framework, which is not applicable to the minimum cooperative unit composed of two UAVs.

Aiming at the minimum cooperative unit, which consists of a known position leader UAV and an unknown position follower UAV, there is little literature on only using the single relative distance to achieve follower UAV high-precision positioning, but there are a few applications in Autonomous Underwater Vehicles (AUVs). Considering the single relative distance between the AUV and a known position beacon, it is difficult to determine an AUV’s spatial position. Many researchers have constructed the virtual beacon arrays as known position beacons to achieve the AUV’s positioning [16,17]. However, the position of the virtual beacon is affected by the beacon calibration error, and the AUV’s velocity error and heading error affect the AUV’s positioning accuracy. In addition, by combining the navigation sensor mounted on the AUV, such as the pressure sensor, Doppler Velocity Log (DVL) and geomagnetic sensor, etc., many researchers have constructed the AUV’s motion model based on motion characteristics. The relative distance or signal propagation time between the known position beacon and AUV is used as the system observation to suppress positioning error divergence of the INS/DVL/pressure-sensor-integrated navigation system. However, due to the lack of sufficient position observation, it can inhibit positioning error accumulation of the integrated navigation system to a certain extent by using the above method. The positioning error of the integrated navigation system is still divergent under long-term conditions [18,19,20,21,22].

Thus, aiming at the minimum cooperative unit, this paper proposes a CP method based on the follower UAV’s moving vector. The error model of the follower UAV’s INS is constructed according to the IMU error characteristic. By combining the output of the speed sensor and magnetic sensor mounted on the follower UAV, the follower UAV’s moving vector is used to construct a position observation equation of the follower UAV’s INS. The improved extended Kalman filter is utilized to estimate the state vector to obtain a follower UAV high-precision navigation solution. In addition, considering that the airborne datalink system based on radio signals may be interfered with by the UAV formation-flying environment, the follower UAV cannot obtain relative distance information with the leader UAV in real time. In this paper, a two-state Markov chain is used to judge the availability of the distance information to improve the efficiency of the CP algorithm. The innovations of this paper are as follows:

1. Aiming at the minimum cooperative unit, which consists of a known position leader UAV and an unknown position follower UAV, this paper proposes a CP algorithm based on follower the UAV’s moving vector.
2. High-precision positioning of the follower UAV is achieved by using the single relative distance information and the follower UAV’s moving vector.
3. Introducing a two-state Markov chain to judge the availability of the distance information improves the efficiency of the cooperative navigation algorithm.

The rest of the paper is organized as follows: In the Section 2, the CP framework and the concept of the follower UAV’s moving vector are introduced. Observation equations based on the follower UAV’s moving vector are established in Section 3. The CP algorithm based on improved extended Kalman filtering is designed in Section 4. In Section 5, the comparative analysis is made between the proposed algorithm and traditional multiple leader UAV CP algorithm based on relative ranging. Finally, the conclusion of this study is summarized in Section 6.

## 2. Overview of the Proposed Cooperative Localization Algorithm

### 2.1. The Framework of Proposed Cooperative Localization Algorithm

**Figure 1:**
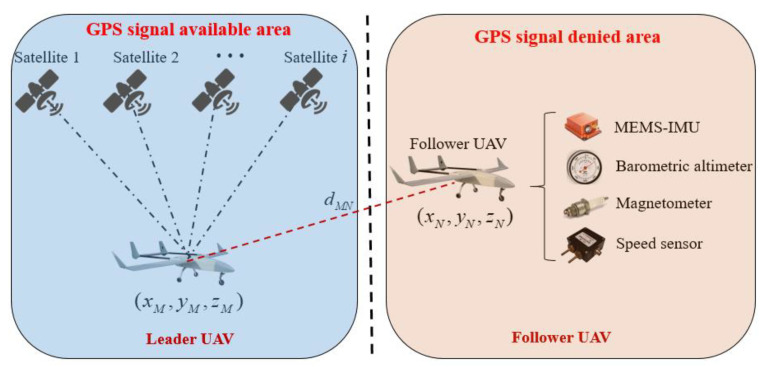
A known position leader UAV and an unknown position follower UAV cooperative localization scenario based on relative ranging.

The CP scenario of the minimum cooperative unit based on relative ranging is shown in Figure 1. In this paper, the geographic coordinate system is defined as the navigation coordinate system. In the navigation coordinate system, with the *x*-axis pointing in the east direction and the *y*-axis pointing in the north direction, the *z*-axis is determined by right-hand rule, and the origin is located at the centroid of the carrier. The front and right directions in the body coordinate system are pointing to the *x*-axis, y-axis, respectively, and the *z*-axis is determined by the right-hand criterion.

**Figure 2:**
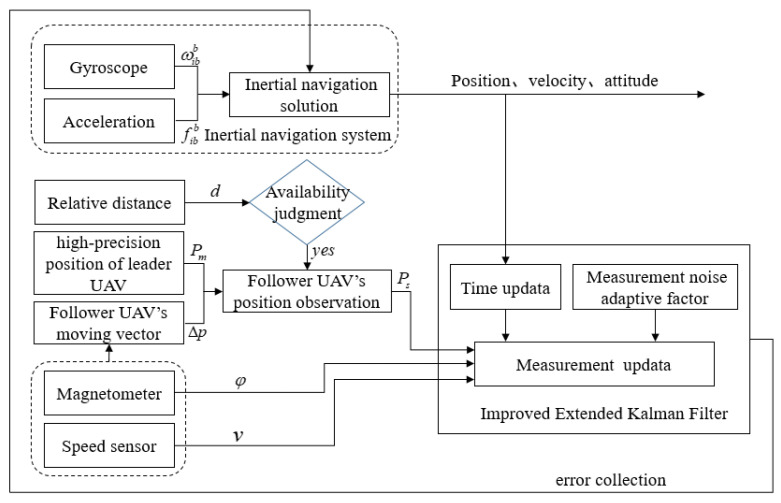
The schematic diagram of proposed method in this paper.

In the above CP scenario, the known position leader UAV flies in an area with available GPS signals and is equipped with a low-precision Micro-Electro-Mechanical System (MEMS) IMU, a GPS receiver in a single-point mode and a datalink communication device. The unknown position follower UAV flies in an area denied GPS signals and carries a low-precision MEMS-IMU, speed sensor, magnetic sensor, barometric altimeter and datalink communication device. The two UAVs perform time calibration by GPS system clock before cooperative flight to ensure each UAV’s time reference is synchronized. In the process of CP, the leader UAV obtains high-precision navigation information by fusing INS/GPS information. Meanwhile, the leader UAV broadcasts its position, speed and course information to the follower UAV node. According to the preset time interval period, the follower UAV receives navigation information transmitted by the leader UAV through the airborne datalink system. Under the condition that the clock of each UAV node in the minimum cooperative unit is synchronized, the relative distance between the leader and follower UAV is obtained in real time by the TOA method. Considering the positioning error of the follower UAV’s INS has accumulated and diverged for a long time, only relying on the positioning accuracy of the follower UAV’s INS is difficult for meeting the UAV cooperative flight requirement. By combining the altitude information obtained from the follower UAV’s barometric altimeter, three-dimensional navigation positioning of the follower UAV is transformed into two dimensions. The moving vector of the follower UAV at adjacent times is constructed using the information of the speed sensor and three-axis magnetic sensor. Based on the error model of the follower UAV’s INS being accurately constructed, the position observation equation of the follower UAV’s INS is constructed using the relative ranging constraint and the follower UAV’s moving vector. Meanwhile, combining the speed and heading information output from the speed and three-axis magnetic sensor, the velocity and heading observation equation of the follower UAV’s INS are constructed, respectively. In addition, considering that the datalink system based on radio signals may be interfered with by the external environment, this paper introduces a two-state Markov chain to judge the availability of the relative distance information. When the distance information among the UAVs is available, the position observation equation of follower the UAV’s INS is constructed by using the follower UAV’s moving vector. Otherwise, only the velocity and heading information obtained from the follower UAV’s airborne sensor are used as the system observation vector. Finally, the improved extended Kalman filtering is designed to estimate the state vector to improve the follower UAV’s positioning accuracy. The schematic diagram of the proposed method in this paper is shown in Figure 2.

### 2.2. Moving Vector of Follower UAV

**Figure 3:**
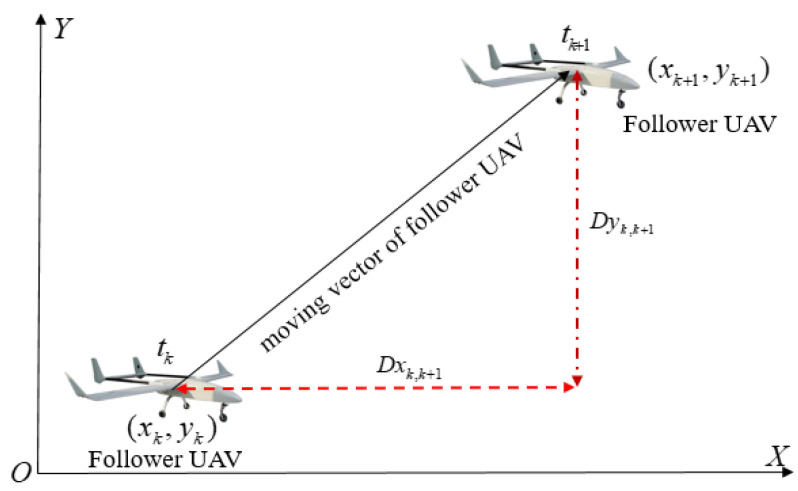
Schematic diagram of follower UAV’s moving vector in the horizontal direction.

Considering there is only one known position leader UAV in the minimum cooperative unit, the single relative distance with the leader UAV cannot determine the follower UAV’s spatial position. The moving vector of the follower UAV has to play an important role in constructing the position observation equation of the follower UAV’s INS. The follower UAV’s moving vector represents the relative displacement vector that the follower UAV removed from time $t_{k}$ to time $t_{k+1}$. The schematic diagram of the follower UAV’s moving vector in the horizontal direction is illustrated in Figure 3. The relative position constraint of the follower UAV from time $t_{k}$ to time $t_{k+1}$ in the horizontal direction can be expressed as:

(1) $$\left[\begin{array}{c} x_{k+1}\\ y_{k+1} \end{array}\right]=\left[\begin{array}{cc} 1 & 0\\ 0 & 1 \end{array}\right]\left[\begin{array}{c} x_{k}\\ y_{k} \end{array}\right]+\left[\begin{array}{cc} 1 & 0\\ 0 & 1 \end{array}\right]\left[\begin{array}{c} Dx_{k,k+1}\\ Dy_{k,k+1} \end{array}\right]=\left[\begin{array}{cc} 1 & 0\\ 0 & 1 \end{array}\right]\left[\begin{array}{c} x_{k}\\ y_{k} \end{array}\right]+\left[\begin{array}{c} \hat{v}\cos\hat{\theta }\cos\hat{\varphi }\Delta t\\ \hat{v}\cos\hat{\theta }\sin\hat{\varphi }\Delta t \end{array}\right]$$

where $Dx_{k,k+1}$ represents the moving vector of the follower UAV in X direction. $Dy_{k,k+1}$ represents the moving vector of the follower UAV in Y direction. $\left(x_{k+1},y_{k+1}\right)$ is the horizontal position of the follower UAV at time $t_{k+1}$. $\left(x_{k},y_{k}\right)$ is the horizontal position of the follower UAV at time $t_{k}$. $\hat{v}$ is the follower UAV’s velocity obtained from the speed sensor. $\hat{\theta }$ is the follower UAV’s pitch angle. $\hat{\varphi }$ is the heading angle obtained from the follower UAV’s three-axis magnetic sensor. Δt is the time interval from time $t_{k}$ to $t_{k+1}$. Considering the existence of sensor measurement error, the follower UAV’s velocity, pitch angle and heading angle have the following form:

(2) $$\left\{\begin{array}{l} \hat{v}=\bar{v}+\Delta v\\ \hat{\theta }=\bar{\theta }+\Delta \theta \\ \hat{\varphi }=\bar{\varphi }+\Delta \varphi \end{array}\right.$$

where $\left(\bar{v},\bar{\theta },\bar{\varphi }\right)$ is the measured true value of velocity, pitch angle and heading angle, respectively, and (Δv,Δθ,Δφ) is the velocity error, pitch angle error and heading error, respectively. Substituting Formula (2) into Formula (1) and ignoring the small error term, the relative displacement error of the follower UAV from time $t_{k}$ to $t_{k+1}$ can be expressed as:

(3) $$\left[\begin{array}{c} \Delta Dx_{k,k+1}\\ \Delta Dy_{k,k+1} \end{array}\right]=\left[\begin{array}{c} -\bar{v}\Delta t(\cos\bar{\theta }\sin\bar{\varphi }\Delta \varphi +\sin\bar{\theta }\cos\bar{\varphi }\Delta \theta )+\Delta v\Delta t(\cos\bar{\theta }\cos\bar{\varphi }-\cos\bar{\theta }\sin\bar{\varphi }\Delta \varphi -\sin\bar{\theta }\cos\bar{\varphi }\Delta \theta )\\ \bar{v}\Delta t(\cos\bar{\theta }\cos\bar{\varphi }\Delta \varphi -\sin\bar{\theta }\sin\bar{\varphi }\Delta \theta )+\Delta v\Delta t(\cos\bar{\theta }\sin\bar{\varphi }+\cos\bar{\theta }\cos\bar{\varphi }\Delta \varphi -\sin\bar{\theta }\sin\bar{\varphi }\Delta \theta ) \end{array}\right]$$

It can be seen from Formula (3) that the moving vector of the follower UAV from time $t_{k}$ to time $t_{k+1}$ in the horizontal direction is jointly affected by the follower UAV’s velocity error, pitch angle error and heading error. Considering the relatively small change of the pitch angle at adjacent times during UAV flight, the relative displacement error of the follower UAV at adjacent times is mainly affected by the velocity error and heading error. Generally, the airborne speed sensor can accurately measure the follower UAV’s velocity, while the three-axis magnetometer is vulnerable to interfere from the external environment. The three-axis magnetometer with temperature and magnetic compensation in advance can obtain higher heading measurement accuracy. The relative displacement accuracy of the follower UAV at adjacent times will affect the follower UAV’s positioning accuracy. The follower UAV’s positioning accuracy can be effectively improved by reducing the relative displacement error of the follower UAV at adjacent times. The smaller relative displacement error of the follower UAV at adjacent times, the higher the follower UAV’s positioning accuracy.

## 3. System Observation Equation Based on Follower UAV’s Moving Vector

**Figure 4:**
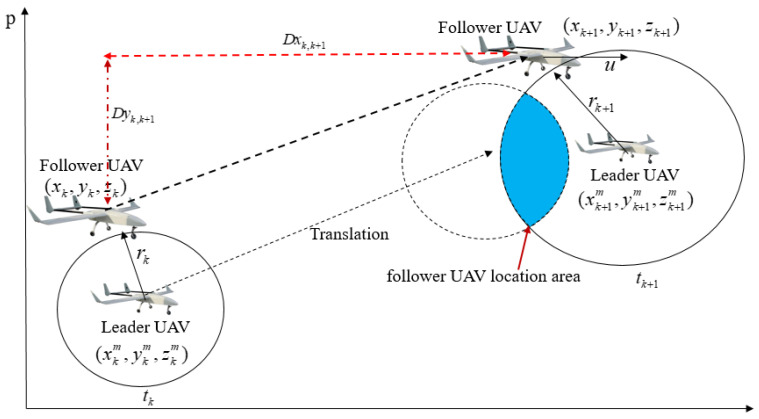
The spatial position relationship in the horizontal direction between leader and follower UAV at adjacent times.

Assuming that each UAV’s clock completes its time synchronization by GPS before UAV formation cooperative flight, in the process of CP, the leader UAV sends its pulse information and position broadcast through the airborne datalink system at time $t_{k}$. Assuming that the $\left(x_{k}^{m},y_{k}^{m},z_{k}^{m}\right)$ and $\left(x_{k}^{},y_{k}^{},z_{k}^{}\right)$ represent the positions of the leader and follower UAV at time $t_{k}$, respectively, the follower UAV receives a pulse signal from the leader UAV and measures relative distance $d_{k}$. Considering the barometric altimeter mounted on the follower UAV can be used to measure altitude information in real time, based on the high-precision position information of the leader UAV and the height information of the follower UAV, the follower UAV’s position solution can be converted from three-dimensional to two-dimensional. The spatial position relationship in the horizontal direction between the leader and follower UAV at the adjacent times is illustrated in Figure 4. We can conclude from Figure 4 that at time $t_{k}$, the follower UAV is located on a circle with $\left(x_{k}^{m},y_{k}^{m}\right)$ as the center and $r_{k}$ as the radius. $r_{k}$ can be expressed as:

(4) $$r_{k}=\sqrt{d_{k}^{2}-{\left(z_{k}^{m}-z_{k}\right)}^{2}}$$

The circular equation at time $t_{k}$ can be expressed as:

(5) $${\left(x_{k}-x_{k}^{m}\right)}^{2}+{\left(y_{k}-y_{k}^{m}\right)}^{2}=r_{k}^{2}$$

where $d_{k}$ is the three-dimensional relative distance between the leader and follower UAV at time $t_{k}$. $z_{k}^{m}$ is the altitude information at time $t_{k}$ obtained from the leader UAV’s INS/GPS loosely integrated navigation system. $z_{k}$ is the altitude information obtained from the follower UAV’s onboard barometric altimeter.

At time $t_{k+1}$, the follower UAV is located on a circle with $\left(x_{k+1}^{m},y_{k+1}^{m}\right)$ as the center and $r_{k+1}$ as the radius. $r_{k+1}$ can be expressed as:

(6) $$r_{k+1}=\sqrt{d_{k+1}^{2}-{\left(z_{k+1}^{m}-z_{k+1}\right)}^{2}}$$

The circular equation at time $t_{k+1}$ can be expressed as:

(7) $${\left(x_{k+1}-x_{k+1}^{m}\right)}^{2}+{\left(y_{k+1}-y_{k+1}^{m}\right)}^{2}=r_{k+1}^{2}$$

where $d_{k+1}$ is the three-dimensional relative distance information between the leader UAV and the UAV at time $t_{k+1}$. $z_{k+1}^{m}$ is the altitude information at time $t_{k+1}$ obtained from the leader UAV’s INS/GPS loosely integrated navigation system. $z_{k+1}$ is the altitude information at time $t_{k+1}$ obtained from the follower UAV’s onboard barometric altimeter. The position solution of the follower UAV at time $t_{k+1}$ can be solved by combining the moving vector of the follower UAV from time $t_{k}$ to $t_{k+1}$ with the circular equation.

(8) $$\left\{\begin{array}{l} {\left(x_{k+1}-Dx_{k,k+1}-x_{k}^{m}\right)}^{2}+{\left(y_{k+1}-Dy_{k,k+1}-y_{k}^{m}\right)}^{2}=r_{k}^{2}\\ {\left(x_{k+1}-x_{k+1}^{m}\right)}^{2}+{\left(y_{k+1}-y_{k+1}^{m}\right)}^{2}=r_{k+1}^{2} \end{array}\right.$$

The first-order linearization Taylor expansion of Equation (8) is made and ignores the higher-order term above the second order, and by combining the altitude information output from the barometric altimeter, the position observation equation of the follower UAV’s INS at time $t_{k+1}$ can be obtained. The position observation equation of the follower UAV’s INS at time $t_{k+1}$ is constructed as follows:

(9) $$\begin{array}{l} \;Z_{k+1}^{p}=H_{k+1}^{p}X_{k+1}+v_{p}\\ \;H_{k+1}^{p}={\left[\begin{array}{ccccc} 0_{3\times 6} & ╎ & H_{3\times 3}^{\rho } & ╎ & 0_{3\times 9} \end{array}\right]}_{3\times 18}\\ H_{3\times 3}^{\rho }\\ =\left[\begin{array}{ccc} -(R_{N}+h)\sin L(\cos\lambda f_{11}+\sin\lambda f_{12}) & -(R_{N}+h)\cos L(\sin\lambda f_{11}-\cos\lambda f_{12}) & \cos L(\cos\lambda f_{11}+\sin\lambda f_{12})\\ -(R_{N}+h)\sin L(\cos\lambda f_{21}+\sin\lambda f_{22}) & -(R_{N}+h)\cos L(\sin\lambda f_{21}-\cos\lambda f_{22}) & \cos L(\cos\lambda f_{21}+\sin\lambda f_{22})\\ 0 & 0 & 1 \end{array}\right] \end{array}$$

where $Z_{k+1}^{p}$ is the position observation vector. $H_{k+1}^{p}$ is the observation Jacobian matrix corresponding to the position measurement equation. $X_{k+1}$ is the state vector of the minimum cooperative unit’s positioning system. $v_{p}$ is the relative distance measurement white noise. (L,λ,h) represents the position information of the follower UAV in the navigation coordinate system. $R_{N}$ represents the curvature radius of the earth in prime vertical. $f_{i1}=\frac{\partial f_{i}}{\partial x},f_{i2}=\frac{\partial f_{i}}{\partial y},i=1,2$ is the direction cosine of the position observation equation in the horizontal direction at time $t_{k}$ and $t_{k+1}$, respectively.

The follower UAV’s onboard speed sensor can acquire the UAV’s forward velocity. The horizontal velocity can be obtained by decomposing the follower UAV’s forward velocity into the horizontal direction. The follower UAV’s horizontal velocity can be expressed as:

(10) $$\left\{\begin{array}{l} v_{x}=\hat{v}\cos\hat{\varphi }\cos\hat{\theta }\\ v_{y}=\hat{v}\sin\hat{\varphi }\cos\hat{\theta } \end{array}\right.$$

In the above formula, $v_{x}$ is the follower UAV’s velocity in x direction. $v_{y}$ is the follower UAV’s velocity in Y direction. Based on Formula (10), the horizontal velocity observation equation of the follower UAV’s INS can be written as follows:

(11) $$\begin{array}{l} Z_{k+1}^{v}=H_{k+1}^{v}X_{k+1}+v_{v}\\ H_{k+1}^{v}={\left[\begin{array}{ccccc} \begin{array}{c} 0_{2\times 3} \end{array} & ┊ & \begin{array}{ccc} 1 & 0 & 0\\ 0 & 1 & 0 \end{array} & ┊ & 0_{2\times 12} \end{array}\right]}_{2\times 18} \end{array}$$

where $Z_{k+1}^{v}$ is the velocity observation vector. $H_{k+1}^{v}$ is the Jacobian matrix corresponding to the velocity measurement equation. $V_{v}$ is the velocity measurement white noise.

At the same time, the follower UAV’s onboard three-axis magnetic sensor can provide heading information for the UAV through a sensitive geomagnetic field, which can be used as the heading observation to correct the error of the follower UAV’s INS. After temperature and magnetic compensation, the heading information output from three-axis magnetic sensor can be expressed as:

(12) $${\hat{\varphi }}_{m}={\bar{\varphi }}_{m}+v_{\varphi }$$

where ${\hat{\varphi }}_{m}$ represents the measured value of the heading angle of the magnetic sensor. ${\bar{\varphi }}_{m}$ represents the true value of heading angle measurement of the magnetic sensor. $v_{\varphi }$ is the magnetic sensor measurement white noise. By combining the heading information calculated by the follower UAV’s INS and the spatial coordinate system’s transformation relationship between the attitude angle and platform misalignment angle, the heading observation equation of the follower UAV’s INS can be constructed by Formula (11) as:

(13) $$\begin{array}{l} Z_{k+1}^{\varphi }=H_{k+1}^{\varphi }X_{k+1}+v_{\varphi }\\ H_{k+1}^{\varphi }={\left[\begin{array}{cc} H_{\varphi }╎ & 0_{1\times 15}╎ \end{array}\right]}_{1\times 18}\\ H_{\varphi }=\frac{1}{\cos\theta }{\left[\begin{array}{ccc} \sin\varphi \sin\theta & \cos\varphi \sin\theta & -\cos\theta \end{array}\right]}_{1\times 3} \end{array}$$

where $Z_{k+1}^{\varphi }$ is the heading observation vector. (θ,φ) represents the pitch angle and heading angle calculated by the follower UAV’s INS, respectively. $H_{k+1}^{\varphi }$ is the Jacobian matrix corresponding to the heading observation equation. $v_{\varphi }$ is the magnetic sensor measurement white noise. Assuming that the relative distance measurement white noise, velocity measurement white noise and heading measurement white noise are independent of each other and satisfy the mean value being zero, the covariance is a fixed value.

(14) $$\left\{\begin{array}{l} E\{v_{p}(k)v_{v}(k)\}=0\\ E\{v_{\varphi }(k)v_{v}(k)\}=0\\ E\{v_{p}(k)v_{\varphi }(k)\}=0 \end{array}\right.\left\{\begin{array}{l} E\{v_{\varphi }{\left(k\right)}^{T}v_{\varphi }(k)\}=R_{\varphi }(k)\\ E\{v_{v}{\left(k\right)}^{T}v_{v}(k)\}=R_{v}(k)\\ E\{v_{p}{\left(k\right)}^{T}v_{p}(k)\}=R_{p}(k) \end{array}\right.$$

## 4. Cooperative Positioning Algorithm Design Based on Improved Extended Kalman Filtering

According to the follower UAV’s INS state recursive equation, more details about the follower UAV’s INS state recursive equation can be found in [23]. The state propagation equation of the improved extended Kalman filtering is constructed as follow:

(15) X(k+1)=f(X(k),k)+w(k)

where

(16) $$\begin{array}{ll} X(k)= & [\varphi_{E}(k),\varphi_{N}(k),\varphi_{U}(k),\delta v_{E}(k),\delta v_{N}(k),\delta v_{U}(k),\delta L(k),\\ & \delta \lambda (k),\delta h(k),\varepsilon_{bx}(k),\varepsilon_{by}(k),\varepsilon_{bz}(k),\varepsilon_{rx}(k),\varepsilon_{ry}(k),\\ & \varepsilon_{rz}(k),\nabla_{ax}(k),\nabla_{ay}(k),\nabla_{az}(k){]}^{T} \end{array}$$

The system state vector X(k) can be, respectively, expressed as: platform misalignment angle error of the follower UAV’s INS, velocity error of the follower UAV’s INS, position error of the follower UAV’s INS, gyroscope random constant drift, gyroscope first-order Markov white noise and accelerometer first-order Markov white noise. w(k) is the system noise input matrix. Assuming that w(k) is the Gaussian white noise with a zero mean and satisfies $E\{w(k)w^{T}(k)\}=Q(k)$, the state transition Jacobian matrix F(k+1,k) and system noise control input Jacobian matrix can be expressed as:

(17) $$\left\{\begin{array}{l} F(k+1,k)=\frac{\partial f(X(k),k)}{\partial X}{|}_{X=\hat{X}(k|k)}\\ W(k+1,k)=\frac{\partial f(X(k),k)}{\partial u}{|}_{X=\hat{X}(k|k)} \end{array}\right.$$

The airborne datalink system based on radio data transmitting and receiving is susceptible to radio signal interference. It is difficult for the follower UAV to receive communication data packets containing relative distance and the leader UAV’s position information at each sampling period. The position observation equation of the follower UAV’s INS has the following form:

(18) $$Z_{k+1}^{P}=\left\{\begin{array}{l} H_{k+1}^{p}X_{k+1}+v_{p}\;\mathrm{if}\;\mathrm{communication}\;\mathrm{packet}\;\mathrm{received}\;\mathrm{successfully}\;\\ 0\;\;\;\;\;\;\;\;\;\mathrm{otherwise}\; \end{array}\right.$$

If the communication data packet from the leader UAV is successfully received at time $t_{k}$, the position observation equation of the follower UAV’s INS can be expressed as $Z_{k+1}^{p}=H_{k+1}^{p}X_{k+1}+v_{p}$. Otherwise, the position observation equation of the follower UAV’s INS is zero. In this paper, in order to improve the follower UAV’s positioning accuracy, the random variable $\gamma_{k}\in \{0,1\}$ is introduced to describe whether the communication data packet arrived at time $t_{k}$. $\gamma_{k}=1$ means that the communication data packet at time $t_{k}$ has been successfully received. In contrast, if $\gamma_{k}=0$ means that the communication data packet at time $t_{k}$ is not successfully received, assuming that the $\gamma_{k}$ follows the two-state Markov chain, the state transition matrix is

(19) $$m=\left[\begin{array}{cc} 1-q & q\\ p & 1-p \end{array}\right]$$

where p,q>0, respectively, indicates the transmission failure and success rate of the communication data packet. The smaller the value of p or the larger the value of q, the better transmission performance of the airborne datalink system. Based on the above definition, the probability distribution of the follower UAV’s position measurement noise $\left\{v_{k},k\geq 1\right\}$ is

(20) $$Ρ(v_{p}|\gamma_{k})\sim \left\{\begin{array}{l} Ν(0,R_{p}),\gamma_{k}=1\\ Ν(0,\sigma^{2}I),\;\gamma_{k}=0 \end{array}\right.$$

where $\sigma^{2}$ is a large positive number. The above formula indicated that if the follower UAV successfully receives the communication data packet at time $t_{k}$, the measurement variance is $R_{p}$. In contrast, the measurement variance is $\sigma^{2}I,\sigma \to \infty$. Due to the velocity and heading observation of the follower UAV’s INS being obtained from onboard sensors, there is no need for data transmission through the airborne datalink system. Therefore, the velocity and heading observation variance of the follower UAV’s INS at time $t_{k}$ are not affected by the datalink system. The position observation of the follower UAV’s INS before time $t_{k}$ is defined as follows:

(21) $$Z_{k}^{p}={\left(Z_{1}^{p},Z_{2}^{p},Z_{3}^{p}\ldots Z_{k}^{p}\right)}^{T}\gamma_{k}={\left(\gamma_{1},\gamma_{2},\gamma_{3},\ldots \gamma_{k}\right)}^{T}$$

Under the condition of the position observation of the follower UAV’s INS being available, the covariance estimation equation of the traditional extended Kalman filtering can be expressed as:

(22) $${\hat{X}}_{k}≜E\{X_{k}|Z_{k}^{p},\gamma_{k}\}$$

(23) $$P_{k}≜E\{(X_{k}-{\hat{X}}_{k}){\left(X_{k}-{\hat{X}}_{k}\right)}^{T}|Z_{k}^{p},\gamma_{k}\}$$

(24) $$P_{k+1/k}≜E\{(X_{k+1}-{\hat{X}}_{k+1/k}){\left(X_{k+1}-{\hat{X}}_{k+1/k}\right)}^{T}|Z_{k}^{p},\gamma_{k}\}$$

(25) $${\hat{Z}}_{k+1/k}^{p}≜E\{Z_{k+1}^{p}|Z_{k}^{p},\gamma_{k}\}$$

However, under the condition of the datalink system being indirectly limited, the following equations are derived from the above formulas:

(26) $$E\{(Z_{k+1}^{p}-{\hat{Z}}_{k+1/k}^{p}){\left(X_{k+1}^{p}-{\hat{X}}_{k+1/k}\right)}^{T}|Z_{k}^{p},\gamma_{k+1}\}=H_{k+1}^{p}P_{k+1/k}$$

(27) $$\begin{array}{l} E\{(Z_{k+1}^{p}-{\hat{Z}}_{k+1/k}^{p}){\left(Z_{k+1}^{p}-{\hat{Z}}_{k+1/k}^{p}\right)}^{T}|Z_{k}^{p},\gamma_{k+1}\}\\ =H_{k+1}^{p}P_{k+1/k}{\left(H_{k+1}^{p}\right)}^{T}+\gamma_{k+1}R_{k+1}^{p}+(1-\gamma_{k+1})\sigma^{2}I \end{array}$$

Due to $X_{k+1/k}$ and $Z_{k+1/k}^{p}$ being independent normal random vectors, its joint probability distribution under the conditions $Z_{k}^{p}$ and $\gamma_{k+1}$ is still following normal distribution and satisfies:

(28) $$E\{X_{k+1/k},Z_{k+1/k}^{p}|Z_{k}^{p},\gamma_{k+1}\}=\left[\begin{array}{c} {\hat{X}}_{k+1/k}\\ H_{k+1}^{p}{\hat{X}}_{k+1/k} \end{array}\right]$$

(29) $$\begin{array}{l} \mathrm{cov}\{X_{k+1/k},Z_{k+1/k}^{p}|Z_{k}^{p},\gamma_{k+1}\}\\ =\left[\begin{array}{c} P_{k+1/k}\;\;\;\;\;\;\;\;\;\;\;\;\;\;\;\;P_{k+1/k}{\left(H_{k+1}^{p}\right)}^{T}\;\\ H_{k+1}^{p}P_{k+1/k}H_{k+1}^{p}p_{k+1/k}{\left(H_{k+1}^{p}\right)}^{T}+\gamma_{k+1}R_{k+1}^{p}+(1-\gamma_{k+1})\sigma^{2}I \end{array}\right] \end{array}$$

Therefore, when the airborne datalink system is indirectly limited, the improved extended Kalman filtering equations are as follows:

(30) $${\hat{X}}_{k+1/k}=F_{k}X_{k}$$

(31) $$P_{k+1/k}=F_{k}P_{k}F_{k}^{T}+Q_{k}$$

(32) $${\hat{X}}_{k+1}={\hat{X}}_{k+1/k}+\gamma_{k+1}K_{k+1}(Z_{k+1}^{p}-H_{k+1}^{p}{\hat{X}}_{k+1/k})$$

(33) $$P_{k+1}=P_{k+1/k}-\gamma_{k+1}K_{k+1}H_{k+1}^{p}P_{k+1/k}$$

(34) $$K_{k+1}=P_{k+1/k}{\left(H_{k+1}^{p}\right)}^{T}{\left(H_{k+1}^{p}P_{k+1/k}{\left(H_{k+1}^{p}\right)}^{T}+R_{k+1}\right)}^{-1}$$

## 5. Multi-UAVs Flight Test and Results

**Figure 5:**
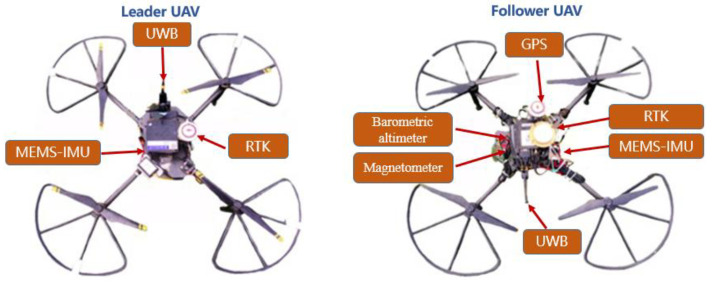
Sensor mounted on the UAVs in autonomous flight experiment.

An outdoor autonomous flight CP experiment involving two UAVs has been carried out on the campus playground. All sensors mounted on the two UAVs are shown in Figure 5. The performance parameters of airborne sensors are shown in Table 1. In the experiment, the leader UAV acquired high-precision position information by an INS/GPS loosely integrated navigation system. The forward velocity output from the follower UAV’s single-point GPS receiver was used to simulate the output of the speed sensor. The ultra-wideband (UWB) sensor was used to realize relative distance measurement in real time. In order to evaluate the follower UAV’s positioning performance, the real-time kinematic (RTK) navigation system mounted on the follower UAV was used as the reference system. The position information output from the RTK is only used as the position reference and does not participate in CP calculation. The three-axis magnetic sensor module with temperature and magnetic compensation and the barometric altimeter module mounted on the follower UAV, respectively, measure the UAV’s heading and altitude information. Before the experiment, the sampling frequency of the follower and leader UAV’s INS were set to 100Hz. The sampling frequencies of the other sensors were all set to 1Hz. In order to compare the positioning performance of the proposed algorithm and the traditional multi-leader UAV CP algorithm based on relative ranging, we made full use of five UWB base stations on the playground to replace the known position leader’s UAV nodes. The positions of the UWB base station were calibrated by GPS in a single-point mode before the experiment. During the experiment, the UWB tag on the follower UAV could obtain relative distance information relative to the UWB base station. Due to the influence of UAV’s motion, the UWB tag mounted on the follower UAV could not obtain relative distance information from the leader UAV at each sampling period. A two-state Markov chain was introduced to judge the availability of the relative distance. When the relative distance information with the leader UAV is available, the position observation equation of the follower UAV’s INS is constructed using the follower UAV’s moving vector. During the experiment, all sensor data were captured from the follower UAV’s processor. The feasibility of the proposed algorithm was verified by post-processing. The total flight time was about 109 s.

**Figure 6:**
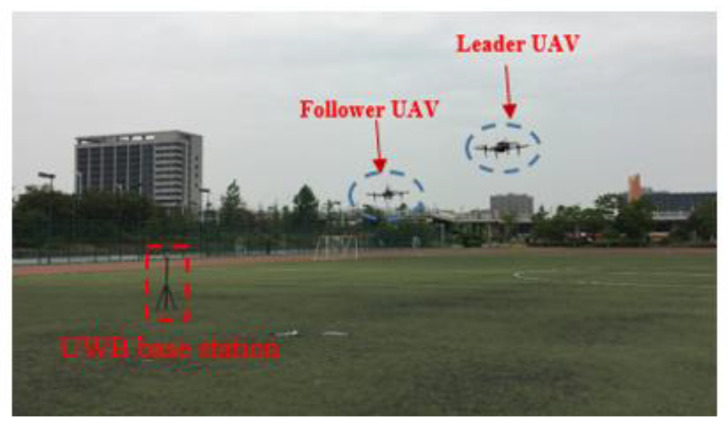
Two UAVs autonomous flight cooperative localization experiment.

**Figure 7:**
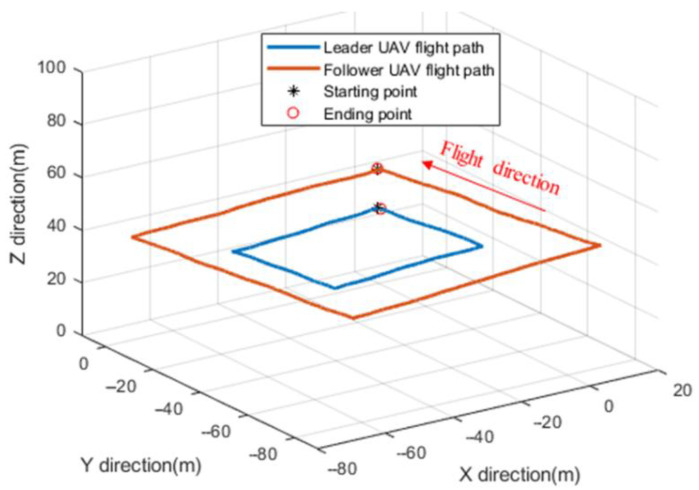
The flight path of leader and follower UAV.

**Figure 8:**
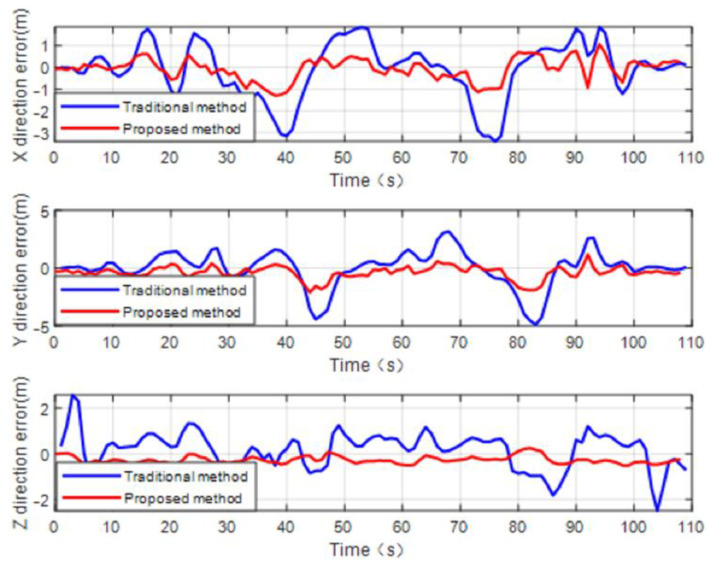
Follower UAV’s positioning error curve comparison by using traditional multi-leader UAV CP method based on relative ranging and the proposed method.

The autonomous flight CP experiment for the two UAVs is shown in Figure 6. The flight path of the two UAVs obtained from the leader and follower UAV navigation processors is shown in Figure 7. Figure 8 illustrates the positioning performance of the follower UAV generated by the traditional multi-leader UAV CP method based on relative ranging and the proposed method.

It can be seen from Figure 8 that during the 109 s flight time, the follower UAV’s positioning error was basically kept within 2 m by using the proposed method. Under the condition that the measurement accuracy of the sensors used in the experiment is consistent, the method proposed in this paper has higher positioning accuracy compared to the traditional multi-leader UAV CP method based on relative ranging.

**Figure 9:**
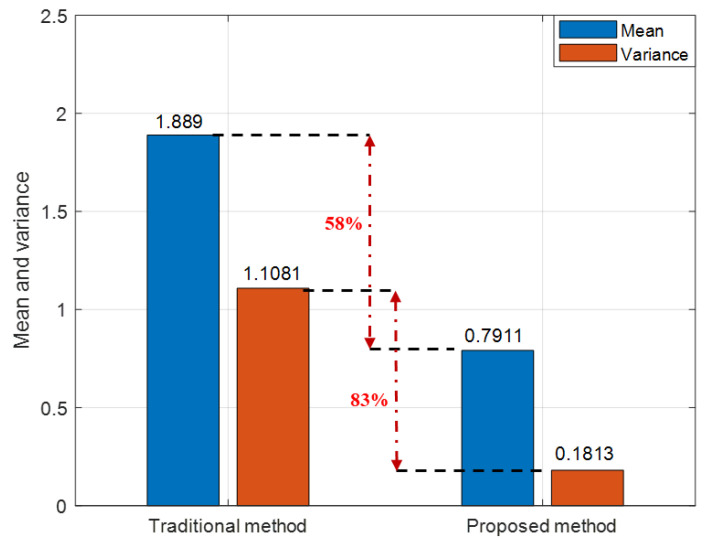
Mean and variance of follower UAV’s positioning error using traditional method and proposed method.

In order to more visually compare the follower UAV’s positioning performance by using the proposed method and the traditional multi-leader UAV CP method based on relative ranging, the root mean square error (RMSE) value of the follower UAV’s positioning error is compared in Table 2. It can be seen from Table 2 that, under the condition that the follower UAV acquires relative distance information from the leader UAV at discontinuous times, the CP algorithm proposed in this paper has higher positioning accuracy than the traditional method. The follower UAV’s positioning accuracy is improved at least 50% compared to the traditional method. For the purpose of quantitatively comparing and analyzing the positioning performance of the proposed algorithm from various perspectives, Figure 9 gives the mean and variance of the follower UAV’s positioning error by using the traditional CP method and the proposed method. We can conclude from the Figure 9 that when there are not enough known position leader UAVs in the UAV cooperative network, the proposed algorithm makes full use of the historical cooperative information to construct position observations. The position closed-form solution of the follower UAV can be acquired in real time. Better results by using the proposed method can be obtained and positioning accuracy can meet the requirement of UAV formation cooperative flight. The results of the analysis based on mean and variance are consistent with the results of the RMSE.

The method proposed in this paper breaks through the limitation of the traditional CP algorithm on the number of known position leader UAV nodes, which lays a theoretical foundation for the future engineering application.

## 6. Conclusions

The traditional multi-leader UAV CP algorithm based on relative ranging has a requirement on the number of known position leader UAV nodes. When the number of leader UAVs and relative distance measurements are limited, the traditional CP algorithm is not applicable. Aimed at the minimum cooperative unit composed of a known position leader UAV and an unknown position follower UAV, this paper proposes a cooperative localization method based on the follower UAV’s moving vector. Compared to the traditional multi-leader UAV CP method based on relative ranging, only the single relative distance information with the leader UAV is required to obtain the follower UAV’s position solution. By combining the information of the follower UAV’s airborne speed sensor and three-axis magnetic sensor, the moving vector of the follower UAV at adjacent times is constructed. The position observation equation of the follower UAV’s INS is constructed by using a relative distance constraint and the follower UAV’s moving vector. Meanwhile, the velocity and heading observation equations of the follower UAV’s INS are constructed by using the information of the speed and magnetic sensor. The improved extended Kalman filtering is designed to estimate and compensate the system state vector to improve the follower UAV’s positioning accuracy. In addition, considering that the datalink system based on radio signals may be interfered with by the external environment, this paper introduces a two-state Markov chain to judge the availability of the relative distance information. The real autonomous flight CP experiment is conducted to validate the performance of the proposed algorithm. The results show that the proposed CP algorithm can achieve better positioning accuracy compared to the traditional multi-leader UAV CP algorithm.

## Figures and Tables

**Table 1:** The performance parameters of airborne sensors in the experiment.

Airborne Sensor Types	Performance Parameters	
Three-axis gyroscope	Bias stability	Random work error
	20 (°/h)	0.2 (${}^{\circ}/\sqrt{h}$)
Three-axis accelerometer	Bias stability	Random work error
	45 µg	10 µg $\sqrt{\mathrm{Hz}}$
Three-axis magnetic sensor	0.5°	
Barometric altimeter	0.5 m	
UWB	0.3 m	
Speed sensor	0.2 m/s	

**Table 2:** Comparison of the RMSE of the follower UAV’s positioning error by using traditional CP method and the proposed method.

Method	Longitude (m)	Latitude (m)	Altitude (m)
Traditional method	1.548	1.274	0.808
Proposed method	0.555	0.505	0.164

## Data Availability

Not applicable.

## References

[1] Sun J., Li B., Jiang Y., Wen C.-Y. (2016). A camera-based target detection and positioning UAV system for search and rescue (SAR) purposes. Sensors.

[2] Liu Y., Liu Z., Shi J., Wu G., Chen C. (2019). Optimization of base location and patrol routes for unmanned aerial vehicles in border intelligence, surveillance, and reconnaissance. J. Adv. Transp..

[3] Lázaro M.T., Castellanos J.A. Localization of probabilistic robot formations in SLAM. Proceedings of the International Robotics and Automation Conference.

[4] Shaferman V., Shima T. (2008). Unmanned aerial vehicles cooperative tracking of moving ground target in urban environments. J Guid Control Dyn..

[5] Raju V.A., Vasundhara P., Reddy V.C.K., Aiswarya A.S. (2018). Improvement of position and orientation of Unmanned Arial Vehicle (UAV) with INS/GPS. Int. J. Eng. Technol..

[6] Guangcai W., Xu X., Zhang T. (2020). M-M Estimation-Based Robust Cubature Kalman Filter for INS/GPS Integrated Navigation System. IEEE Trans. Instrum. Meas..

[7] Outamazirt F., Fu L., Lin Y., Abdelkrim N. (2016). A new SINS/GPS sensor fusion scheme for UAV localization problem using nonlinear SVSF with covariance derivation and an adaptive boundary layer. Chin. J. Aeronaut..

[8] Khalaf W., Chouaib I., Wainakh M. (2017). Novel adaptive UKF for tightly coupled INS/GPS integration with experimental validation on an UAV. Gyroscopy Navig..

[9] Huang S., Huang J., Tang D., Chen F. Research on UAV flight performance test method based on dual antenna GPS/ins integrated system. Proceedings of the 3rd International Communication and Information Systems (ICCIS) Conference.

[10] Cao S., Qin H., Cong L., Huang Y. (2021). TDMA Datalink Cooperative Navigation Algorithm Based on INS/JTIDS/BA. Electronics.

[11] Duan Q. (2014). A High Precision Time Synchronization Scheme for Avionics System. Telecommun. Eng..

[12] Ranger J.F.O. (1996). Principles of JTIDS relative navigation. J. Navig..

[13] Yang C., Strader J., Gu Y., Hypes A., Canciani A., Brink K. Cooperative UAV navigation using Inter-Vehicle Ranging and Magnetic Anomaly Measurements. Proceedings of the 2018 AIAA Guidance, Navigation and Control Conference.

[14] Qu Y., Zhang Y. Cooperative localization of low-cost UAV using relative range measurements in multi-UAV flight. Proceedings of the AIAA Guidance, Navigation and Control Conference.

[15] Chen M., Xiong Z., Liu J., Wang R., Xiong J. (2020). Cooperative navigation of unmanned aerial vehicle swarm based on cooperative dilution of precision. Int. J. Rob. Syst..

[16] Jun C., Zheng C., Sun D., Zhang D. (2017). AUV positioning based on single-beacon ranging in straight-line trajectory. J. Harbin Eng. Univ..

[17] Fangfang C., Weidong L., Juanli L. (2011). Navigating and positioning based on EKF for unmanned underwater vehicle from a single beacon. Comput. Meas. Control.

[18] Zhang F.B., Zhang Y.Q. (2012). Correcting localization error with a single beacon for AUV. Torpedo Technol..

[19] Kepper J.H., Claus B.C., Kinsey J.C. (2018). A Navigation Solution Using a MEMS IMU, Model-Based Dead-Reckoning, and One-Way-Travel-Time Acoustic Range Measurements for Autonomous Underwater Vehicles. IEEE J. Ocean. Eng..

[20] Deng Z.C., Yu X., Qin H.D., Zhu Z.B. (2018). Adaptive Kalman Filter-Based Single-Beacon Underwater Tracking with Unknown Effective Sound Velocity. Sensors.

[21] Sun J., Hu F., Jin W., Wang J., Wang X., Luo Y., Yu J., Zhang A. (2020). Model-aided localization and navigation for underwater gliders using single-beacon travel-time differences. Sensors.

[22] Qin H.D., Yu X., Zhu Z.B., Deng Z.C. (2020). An expectation-maximization based single-beacon underwater navigation method with unknown ESV. Neurocomputing.

[23] Xu J., Xiong Z., Liu J., Wang R. (2019). A dynamic vector-formed information-sharing algorithm based on two-state chi square detection in an adaptive federated filter. J. Navig..

